# Surgical and endovascular revascularization of chronic mesenteric ischemia

**DOI:** 10.1007/s00423-022-02462-3

**Published:** 2022-02-19

**Authors:** Steffen Wolk, Marvin Kapalla, Stefan Ludwig, Christoph Radosa, Ralf-Thorsten Hoffmann, Jürgen Weitz, Christian Reeps

**Affiliations:** 1 Department of Visceral, Thoracic and Vascular Surgery, Universitätsklinikum Carl Gustav Carus, Technische Universität Dresden, Dresden, Germany; 2 Institute for Diagnostic and Interventional Radiology, Universitätsklinikum Carl Gustav Carus, Technische Universität Dresden, Dresden, Germany

**Keywords:** Chronic mesenteric ischemia, Endovascular treatment mesenteric ischemia, Surgical treatment mesenteric ischemia

## Abstract

**Purpose:**

Chronic mesenteric ischemia (CMI) is a rare but life-threatening disease. This study reviewed outcomes in patients treated surgically for CMI by open treatment (OT) and endovascular treatment (ET), analyzing risk factors for endovascular failure.

**Methods:**

Clinical data for 36 patients treated for CMI from 2007 to 2017 were retrospectively analyzed. The study’s primary endpoint was symptom-free survival. The secondary endpoint was the primary technical success for endovascular and open surgical treatments. Risk factors for endovascular failure were identified by using univariate analysis.

**Results:**

Patients were analyzed as treated: 21 patients (58.3%) in the ET and 15 (41.6%) in the OT group. Overall, 20 patients (56%) presented with abdominal angina, 9 (25%) with rest pain, and 7 (19%) without symptoms. An ET was initially attempted in 31 patients (86.1%). The conversion rate from ET to OT was 32.3%, which resulted in a primary technical success of 67.6% in ET and 100% in OT. Six patients from the ET group (19.3%) required surgical revision due to restenosis. One-year (OT 91.6% vs. ET 96.8%; *n.s.*) and three-year primary patency (OT 91.6% vs. ET 80.6%; *n.s.*) as well as 3-year symptom-free survival did not differ between the groups (OT 62.5% vs. ET 69.4%; *n.s*). Overall, in-hospital mortality was 2.8% (*n* = 1), which was not statistically different between the groups (OT 6% vs. ET 0%; *n.s.*). High-grade stenosis of the superior mesenteric artery tended to be associated with higher technical failure (*P* = 0.06).

**Conclusions:**

ET showed a comparable perioperative outcome with higher technical failure. OT was distinguished by excellent early and late technical success.

## Introduction

Chronic mesenteric ischemia (CMI) is a severe disease characterized by postprandial pain and weight loss and occurs when visceral vessels develop high-grade stenosis or occlusion [1, 2]. Early therapy for the symptomatic disease is important to prevent cachexia and end-organ ischemia, associated with high mortality of 50–69% [3, 4]. The clinical presentation of chronic occlusive visceral vessel disease can be asymptomatic or nonspecific over a long period because of the extensive collateral network of the visceral vessels and can delay the diagnosis of acute-onset CMI. Park et al. reported that only 43% of patients with acute mesenteric ischemia had previous symptoms of CMI [5]. In light of this, a medium time to diagnosis in patients with symptomatic CMI of 35 months, as reported by Sandman et al., seems unacceptably long and may have devastating consequences [6]. Because of advances in diagnostic imaging and endovascular ability, diagnosis and treatment of CMI have increased during recent decades [7, 8] Nevertheless, according to European Society for Vascular Surgery (ESVS) guidelines, revascularization is still recommended only in patients who develop symptoms of CMI. Meanwhile, the endovascular approach replaced the open surgical approach, the standard for many years, as first-line treatment [9, 10]. Based on current best available evidence, endovascular revascularization reportedly with lower perioperative mortality and morbidity, but it is also associated with a higher rate of restenosis requiring interventions and recurrent symptoms. The differences in long-term survival between these two treatment modalities remain unclear [9, 11]. Because most comparative series are limited by preselection bias or lack of long-term follow-up, the quality of the supporting data is still restricted. Hence, the purpose of this study was to review outcomes in patients treated for CMI with an open surgical or endovascular approach and to determine symptom-free survival.

## Methods

### Data collection and study population

All consecutive patients with chronic mesenteric ischemia treated by an open surgical or an endovascular approach between 01/2007 and 03/2020 were prospectively recorded in the Department of Visceral, Thoracic and Vascular Surgery at the Carl Gustav Carus University Hospital, Dresden. The data for each case were analyzed retrospectively based on electronic patient records. Inclusion criteria were a diagnosis of CMI by classic symptoms (abdominal pain, postprandial pain, weight loss) and radiologic evidence of high-grade stenosis (> 70%) or occlusion of at least one mesenteric vessel. Exclusion criteria were acute mesenteric ischemia and incomplete records. Demographics, comorbidities, clinical presentation, laboratory parameters, radiologic data, treatment modalities, complications, length of hospital stay, and follow-up examinations were collected.

### Ethics approval

All procedures in studies involving human participants complied with the ethical standards of the institutional research committee.

Under the guidelines for research on human subjects, the local ethics committee at the Technische Universität Dresden approved the study (decision number EK 26,012,018). The ethics committee is registered as institutional review board (IRB) at the Office for Human Research Protections (OHRP) (registration number (IRB00001473 and IORG0001076).

### Indications and surgical/interventional technique

Indications for revascularization were set in the presence of characteristic clinical symptoms of CMI and a ≥ 70% visceral vessel stenosis verified by duplex sonography and computed tomography angiography (CTA) or magnetic resonance angiography (MRA) [12] and after exclusion of other differential diagnoses by a gastroenterologist. An interdisciplinary vascular board (radiologists, angiologists, vascular surgeons) decided whether patients underwent open or endovascular treatment based on the patients’ anatomy, clinical status, and surgical risk factors with a strong trend toward an endovascular first approach. Open treatment included arterial bypass or thrombendarterectomy. Access was created via a midline laparotomy. Depending on the pathology and anatomic conditions a retrograde (iliaco-mesenteric, iliaco-celiac) or antegrade (aorto-celiac + aorto-mesenteric) bypass has been established. In the case of thrombendarterectomy, a venous patch angioplasty was performed. Patients underwent ET by an interventional radiologist under local anesthesia. Percutaneous transfemoral or transbrachial access was used depending on the angle between the visceral arteries and the aorta. After a 4-French sheath was introduced using the Seldinger technique into the femoral or brachial artery, the abdominal aorta was catheterized to perform an aortography to localize the ostium of the target vessel. Balloon-mounted bare-metal stents were used (Express™ LD, Boston Scientific; Marlborough, MA, USA, and Astron Pulsar ®; BIOTRONIK SE & Co. KG, Germany). A long 5- to 8-French sheath (depending on the type and diameter of the stent) was introduced into the celiac trunk or the superior mesenteric artery and a 0.035″ stiff guidewire was placed into a peripheral branch of the artery. After determination of the adequate diameter and length in the peri-interventional angiography, the stent was placed into the vessel and released.

Technical success was defined as a ≤ 30% residual stenosis by angiography. Technical success was defined on duplex sonography as peak systolic velocity of less than a threshold of 3 m/s and without post-stenosis signal. A high-grade stenosis was defined by a PSV > 3 m/s and post-stenosis signal.

All patients were loaded before the intervention with 300 mg clopidogrel and received antiplatelet therapy after the procedure. After stent implantation, patients received dual antiplatelet therapy with acetylsalicylic acid (ASA) 100 mg and clopidogrel 75 mg for 6 weeks, then ASA 100 mg daily as lifelong monotherapy. Routine follow-up consisted of clinical examination and duplex sonography every 3 and 6 months during the first year and at least annually after that.

### Outcome parameters and definitions

The primary endpoint of this study was symptom-free survival. Secondary outcome parameters were primary technical success, morbidity, in-hospital mortality, nutrition score (nutritional risk screening (NRS)) [13], patency rates, and overall survival. All re-interventions/reoperations after primary successfully treated vessel were denoted as vascular revisions. The early postprocedure period was defined as the first 30 days after treatment or during hospital stay if the length was more than 30 days. The follow-up period was the period from hospital discharge until the last available clinical examination. Patency rates were classified into primary, assisted primary, and secondary patency. According to Rutherford et al., primary openness is defined as uninterrupted patency (time from the primary intervention to the next intervention or to the endpoint if no re-intervention was necessary). Secondary patency would be between secondary intervention and the endpoint if further interventions were necessary due to complete closure. Assisted primary patency described the time between the re-intervention and the endpoint, if prophylactic re-interventions were necessary without a complete occlusion [14]. Risk factors for endovascular failure were identified by using univariate analysis.

### Statistical analysis

Statistical analysis was performed using IBM SPSS for Windows, Version 21.0 (IBM Corp., Armonk, NY). All clinical characteristics were grouped to build categorical or nominal variables. Patients were analyzed as treated in two groups: endovascular treatment (ET) and open treatment (OT). Dichotomous variables were recorded as absolute frequencies (number of cases) and relative frequencies (percentages). Continuous data are presented as mean and standard deviation, non-symmetrical with median, and interquartile range (IQR). Pearson’s chi-squared or Fisher’s exact test was used to analyze categorical variables. Differences between means were tested with *t*-test or Mann–Whitney *U* test. Survival and patency data were analyzed using the log-rank test. A two-sided *P*-value < 0.05 was considered statistically significant.

## Results

### Study population and patient characteristics

**Fig. 1:**
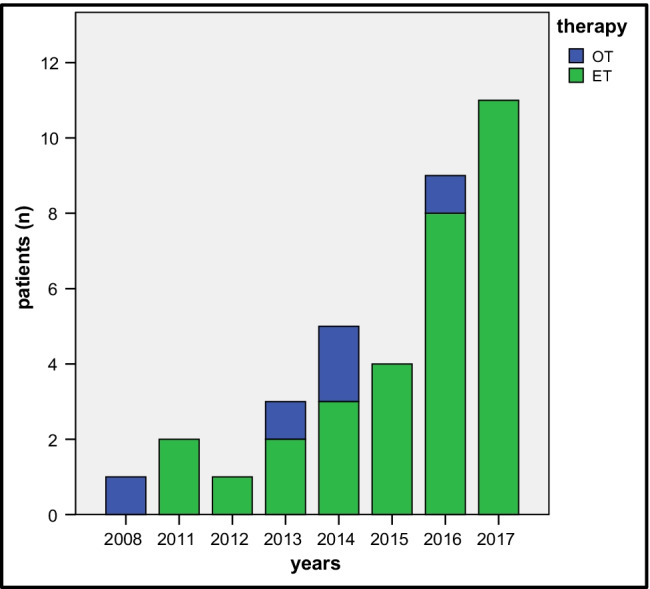
Number of patients with chronic mesenteric ischemia by treatment method from 2007–2017; ET, endovascular treatment; OT, open treatment

The study included 36 patients (male *n* = 22, 61%) with a mean age of 68.9 ± 11.4 years. The therapy of CMI was endovascular treatment (ET) in 31 patients (36.1%) and open surgical treatment (OT) in 5 patients (13.9%). During the study period, the percentage of ET increased perceptibly from 0% in 2008 up to 82% in 2017 (Fig. 1). A total of 55.6% of patients had the concomitant peripheral occlusive arterial disease (POAD) (> Fontaine stage II) and 63.9% had prior vascular surgery or intervention. Other common comorbidities and risk factors were hypertension (91.7%), nicotine abuse (50%), chronic kidney disease (glomerular filtration rate (GFR) < 30 ml/min/1.73m^2^) (33.3%), diabetes mellitus (30.6%), and congestive heart failure (New York Heart Association (NYHA) II) (16.7%). In the nutritional risk screening, a score over 3 (indication of manifest malnutrition) was determined in 50% of the patients (Table 1).

**Table 1:** Demographic and clinical data (intention-to-treat analysis)

Variable*	OT	ET	∑
	*n* = 5 (%)	*n* = 31 (%)	*n* = 36 (%)
*Demographic data*			
Age (years)	69.4 ± 9.9	68.8 ± 11.7	68.9 ± 11.4
Sex (male/female)	3/2 (60/40)	19/12 (61/39)	22/14 (61/39)
*Risk factors and comorbidities*			
BMI (kg/cm^2^)	21.2 ± 5.3	23.9 ± 4.5	23.5 ± 4.6
Chronic kidney disease**	2 (40)	10 (32)	12 (33)
Heart failure (> NYHA II)	-	6 (19)	6 (17)
Atrial fibrillation	1 (20)	6 (19)	7 (19)
Hypertension	4 (80)	29 (94)	33 (92)
CHD	1 (20)	10 (32)	11 (31)
PAOD (> Fontaine stage II)	1 (20)	19 (61)	20 (56)
Myocardial infarction	1 (20)	5 (16)	6 (17)
Hyperlipidemia	3 (60)	21 (67)	24 (66)
Diabetes mellitus	-	11 (36)	11 (31)
COPD	-	6 (19)	6 (17)
Nicotine abuse	2 (40)	16 (52)	18 (50)
Alcohol abuse	-	1 (3)	1 (3)
Previous vascular surgery	3 (60)	20 (65)	23 (64)
NRS score (> 3)*	4 (80)	14 (45)	18 (50)

*OT*, open treatment; *ET*, endovascular treatment; *BMI*, body mass index; *CHD*, coronary heart disease; *COPD*, chronic obstructive pulmonary disease; *PAOD*, peripheral arterial occlusive disease

Continuous data presented as mean ± standard deviation

^*^Nutritional risk screening

^**^GFR < 30 ml/min/1.73m^2^

### Clinical presentation and treatment of CMI

Overall, chronic abdominal discomfort was present in 30 patients (83.3%), weight loss (> 10% of body weight in six months) in 23 patients (63.9%), diarrhea and vomiting in 14 (38.8%), and six (16.7%) patients, respectively.

A clinical presentation with postprandial pain (abdominal angina) was found in 20 patients (55.6%) and rest pain was present in nine patients (25%). Treatment of asymptomatic patients (19.4%) was based on significant three-vessel diseases or poorly collateralized two-vascular occlusions in preparation for an aortic aneurysm repair after careful risk–benefit consideration.

In total, 72 vessels were occluded. Six patients (17%) had one-vessel, 24 (67%) two-vessel, and six (17%) three-vessel disease. The superior mesenteric artery (SMA) was affected in 94.4%, the celiac trunk (CT) in 83.3%, and the inferior mesenteric artery (IMA) in 16.7% of the patients (Table 2). Further radiographic findings before treatment were intestinal wall thickenings in eight patients (22.2%) and extensive calcification of the aorta in 24 (77%).

**Table 2:** Vessels

Variable*	OT	ET	∑
	*n* = 5 (%)	*n* = 31 (%)	*n* = 36 (%)
*Extension of disease*			
Single-vessel disease	-	6 (19)	6 (17)
Two-vessel disease	3 (60)	21 (68)	24 (67)
Three-vessel disease	2 (40)	4 (13)	6 (17)
∑	12	60	72
CT	5 (100)	25 (81)	30 (83)
SMA	5 (100)	29 (94)	34 (94)
IMA	2 (33)	4 (13)	6 (17)
*Vessel revascularization*			
Single-vessel revascularization	2 (40)	17 (55)	19 (53)
Two-vessel revascularization	3 (60)	13 (42)	16 (44)
Three-vessel revascularization	0	1 (3)	1 (3)
∑	8	46	54

*OT*, open treatment; *ET*, endovascular treatment; *SMA*, superior mesenteric artery; *CT*, coeliac trunk; *IMA*, inferior mesenteric artery

^*^Continuous data presented as mean ± standard deviation

Significant differences in the preoperative laboratory parameters were observed with lower serum protein concentrations with progressive stages of disease (postprandial pain: 67 g/l [55–71 g/l] vs. rest pain: 49 g/l [40–52 g/l]; *P* = 0.03). Serum lactate concentration did not differ significantly between stages of disease (postprandial pain: 1.5 mmol/l [0.9–2.6 mmol/l] vs. rest pain: 2 mmol/l [1–3.2 mmol/l]; *P* = 0.82) (Tables 3 and 4).

**Table 3:** Endovascular failure

Variable*	Failure	Success	∑	P
	n = 10 (%)**	n = 21 (%)**	n = 31 (%)**	
Aortic calcification	7 (70)	17 (81)	24 (77)	.4
*Access*				
Femoral	4 (40)	9 (43)	13 (42)	.56
Brachial	6 (60)	10 (48)	16 (52)	.56
Calcification access	4 (40)	6 (40)	10 (44)	.49
*Truncus coeliacus*	7 (70)	18 (86)	25 (81)	.3
Takeoff angle (°)	54 ± 16	57 ± 17	56 ± 16	.69
Closure begin (mm)	2.5 ± 4.3	2.8 ± 4.3	2.8 ± 4.2	.67
< 20 mm	7 (100)	18 (100)	25 (100)	-
> 20 mm	-	-	-	-
Occlusion lengths (mm)	11.4 ± 7.1	13.1 ± 8.5	12.6 ± 8	.61
< 20 mm	6 (86)	13 (77)	19 (79)	.61
> 20 mm	1 (14)	4 (23)	5 (21)	.61
Degree of stenosis (%)	83 ± 16	85 ± 14	84 ± 15	.68
50–70%	1 (14)	3 (17)	4 (16)	.95
> 70%	4 (57)	9 (50)	13 (52)	.95
Occlusion	2 (29)	6 (33)	8 (32)	.95
*A. mesenteric superior*	9 (90)	20 (95)	29 (94)	.58
Takeoff angle (°)	33 ± 15	34 ± 16	34 ± 16	.69
Closure begin (mm)	4.1 ± 5.3	4.8 ± 5.1	4.6 ± 5.1	.77
< 20 mm	9 (100)	19 (100)	28 (100)	-
> 20 mm	-	-	-	-
Occlusion lengths (mm)	20.4 ± 11.5	18.5 ± 8.3	19.1 ± 9.3	.76
< 20 mm	5 (56)	9 (47)	14 (50)	.69
> 20 mm	4 (44)	10 (53)	14 (50)	.69
Degree of stenosis (%)	95 ± 10	87 ± 12	89 ± 12	.06
50–70%	-	1 (5)	1 (3)	.09
> 70%	2 (22)	12 (60)	14 (48)	.09
Occlusion	7 (78)	7 (35)	14 (48)	.09

*SMA*, superior mesenteric artery; *CT*, coeliac trunk; *IMA*, inferior mesenteric artery

^*^Continuous data presented as mean ± standard deviation

^**^Percentages in each group

**Table 4:** Postoperative course and outcomes (as-treated analysis)

Variable*	OT	ET	∑
	*n* = 15 (%)	*n* = 21 (%)	*n* = 36 (%)
*Complications*			
Acute kidney injury	-	1 (3)	1 (3)
Major bleeding (hb-relevant)	2 (13)	-	2 (6)
Wound healing disorder	1 (7)	-	1 (3)
Sepsis	1 (7)	-	1 (3)
Aneurysm spurium	-	2 (6)	2 (6)
Reclosure	-	1 (3)	1 (3)
NSTEMI	1 (7)	-	1 (3)
*Outcomes*			
Perioperative mortality	1 (7)	-	1 (2.8)
Hospital length of stay (days)	21 ± 11	11 ± 10	12 ± 10
Intensive care unit stay (days)	3 ± 4	1 ± 2	2 ± 2

*NSTEMI*, non-ST-elevation myocardial infarction; *OT*, open treatment; *ET*, endovascular treatment

^*^Continuous data presented as mean ± standard deviation or median and range

**Fig. 2:**
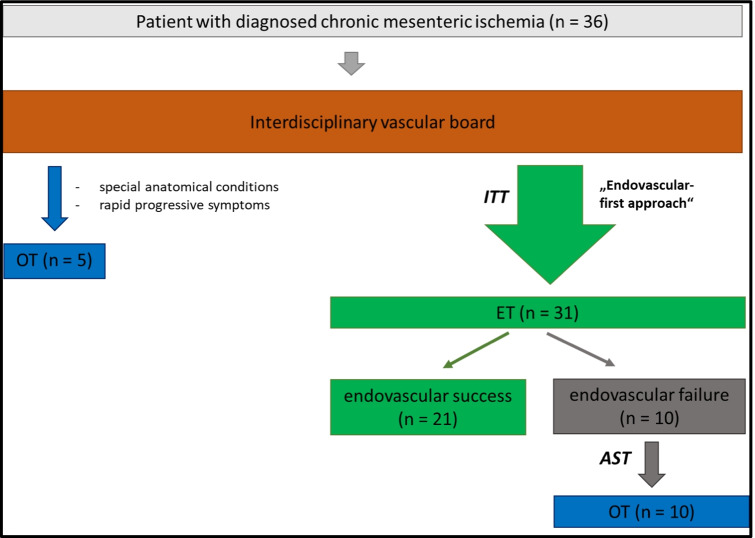
Flowchart therapy method by disease stage; ET, endovascular treatment; OT, open treatment; ITT, intention-to-treat; AST, as-treated

In the ET group (*n* = 31), 21 patients underwent successful stent angioplasty (67.7%); ten endovascular intervention attempts (32.3%) failed before revascularization, either because the mesenteric vessel orifice could not be entered, or the target lesion could not be passed. In the OT group (*n* = 5), four patients received bypass surgery and one patient thromboendarterectomy with venous patch angioplasty (Table 5, Fig. 2).

**Table 5:** Type of bypass

Variable*	Initial OT	Unsuccessfull ET	∑
	*n* = 4 (%)	*n* = 10 (%)	*n* = 14 (%)
*Antegrade*	1 (25)	5 (50)	6 (43)
Aorto-mesenteric	-	4 (80)	4 (67)
Aorto-coeliac	-	-	-
Aorto-mesenteric + coeliac	1 (100)	1 (20)	2 (33)
*Retrograde*	3 (75)	5 (50)	8 (57)
Iliaco-mesenteric	2 (67)	4 (80)	6 (75)
Iliaco-coeliac	-	-	-
Iliaco-mesenteric + coeliac	1 (33)	1 (20)	2 (25)

*OT*, open treatment; *ET*, endovascular treatment

The type of bypass in initial surgery was retrograde in three patients (iliaco-mesenteric + iliaco-coeliac; iliaco-mesenteric (SMA + IMA); iliaco-mesenteric (SMA)), and antegrade in one case (aorto-coeliac + aorto-mesenteric). There was no significant correlation between the extent of aortic calcification and the type of bypass performed. As material PTFE, grafts were used in *n* = 9 (64%) and autologous veins *n* = 5 (36%) cases. All patients treated unsuccessfully with ET subsequently underwent open revascularization. The endovascular failure showed no statistical influence on patient survival.

In summary, 56 vessels were treated, single-vessel revascularization in 19 patients (53%), two vessels in 16 patients (44%), and all three vessels in one patient (3%).

### Patient outcome

**Fig. 3:**
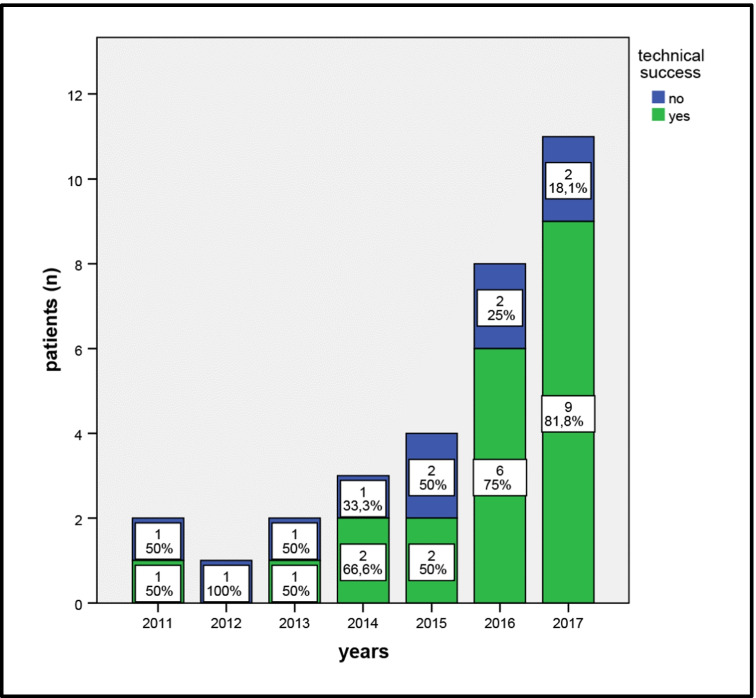
Technical success for endovascular treated patients from 2007–2017

Technical success was achieved in 21 of 31(67.7%) endovascular treated patients and in all primary open procedures (*n* = 5; 100%; *P* = 0.03). One endovascular successfully treated patient required an open surgical vascular revision due to acute stent occlusion. Additionally, ten patients underwent secondary OT after a failed endovascular intervention. In the final as-treated analysis, 21 patients after ET and 15 after OT were included (Fig. 2). In our cohort, we see an increase of endovascular interventions during our study period with a trend to an improved technical success since 2011 (Fig. 3).

Morbidity (medical problems caused by a treatment) in our total study cohort was 22.2% (Table 4). The most common complications were bleeding (5.6%), acute kidney injury (3%), and septic multi organ failure (3%). Major complications (> Clavien-Dindo 3b) occurred significant more after OT compared to ET (7% versus 3%, *P* < 0.02). Overall, in-hospital mortality was 2.8% (*n* = 1) and did not differ significantly between treatment groups (OT 6.7%, *n* = 1 vs. ET 0%, *n* = 0; *P* = 0.42). Hospital stay was significantly reduced in the ET group (11 ± 10 days vs. 21 ± 11 days; *P* < 0.01).

The mean follow-up period for the entire study population was 43 months (range 33–55 months). Overall, 32 of 35 patients could be followed up (91.4%) and three patients were lost in follow-up.

The 3-year primary patency was 100% in the OT and 76.5% in the ET group with a statistical tendency for OT treatment (*P* = 0.06).

**Fig. 4:**
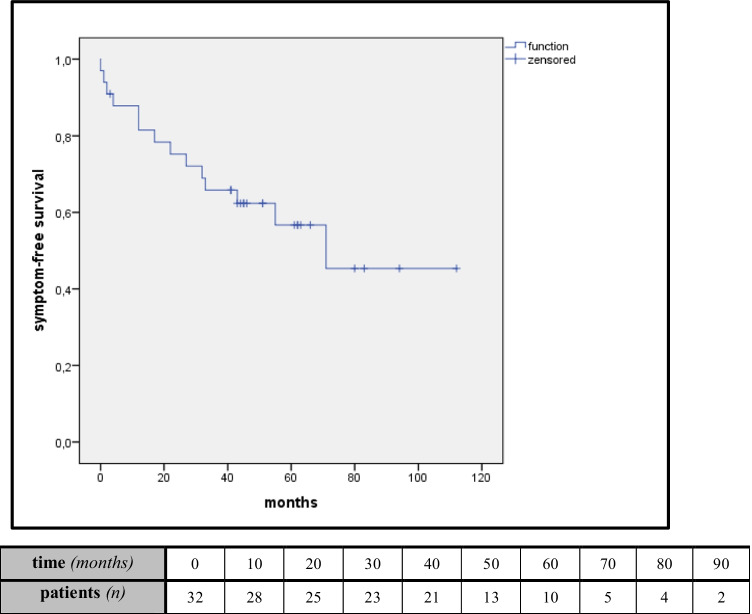
Kaplan–Meier estimates of symptom-free survival and patients at risk

Six successfully endovascularly treated patients (19.4%) required surgical revision due to recurrent symptoms. Only one distal anastomosis stenosis (8.3%) after 29 months of follow-up occurred in an open surgical revascularized patient, who were treated with an antegrade reversed vena saphena magna bypass to the SMA. In the absence of clinical symptoms and low-grade stenosis verified by duplex sonography without kinking of the graft, this was not classified as requiring treatment. There were 13 late deaths (37.1%), which were not related to mesenteric ischemia or complications. Estimated Kaplan–Meier 5-year symptom-free survival analysis showed no difference between the groups (OT 61.5% vs. ET 53.6%; *P* = 0.75, Fig. 4)*.* Two-vessel revascularizations showed no better symptom-free survival than one-vessel revascularization (*P* = 0.75).

### Risk factors for endovascular failure

Univariate analysis identified no significant influence of the manner of access way (transbrachial vs. transfemoral) or extension of the aortic calcification on endovascular failure. A higher degree of stenosis of the superior mesenteric artery (SMA) tended to be associated with a higher probability of endovascular failure (95% ± 10% vs. 87% ± 12%; *P* = 0.06). Overall, seven of the 14 occlusions of the SMA could not be successfully treated endovascularly (*P* = 0.09). No significant influence was observed regarding the angle of takeoff, the closure begin or the length of the SMA, and coeliac trunk occlusions (Table 3).

## Discussion

In our center, the “endovascular first approach” has become the method of choice for “early stage” patients with CMI because of the lower periprocedural major morbidity and the shorter length of hospital stay. Nevertheless, there was a technical success in only 67.7% of the patients because of the impossibility of target vessel recanalization due to highly calcified stenosis. During the study period, we see an evolution of CMI treatment by the endovascular approach with better technical success rates compared to early areas (Fig. 3). All interventions were performed by three experienced radiologists, who are trained in different visceral artery interventions in our high-volume tertiary center (e.g., aneurysm and pseudoaneurysm treatment, hemorrhage treatment). Furthermore, beside the benefits of improved technical success, lower length of hospital stay, and morbidity by ET, OT was offered as backup complementary to ET treatment by a 24 h/7 days vascular surgery service in every patient.

As mentioned in the current guidelines from the European Society for Vascular Surgery, “the superior long-term results of open surgery must be offset against a possible early benefit of endovascular intervention concerning periprocedural mortality and morbidity” (class 1, level of evidence B) [8]. Therefore, open surgical revascularization will still play an important role. Thus, the technical success rate of ET may be lower than described in other studies, with success rates from 87 to 97% [15, 16]. Zacharias et al. identified heavily calcified visceral aortas as well as proximal (< 2 cm from the mesenteric vessel takeoff) and long lesions (> 2 cm) to cause endovascular failure [1]. In our analysis, neither long-stretch lesions (> 2 cm) nor occlusions close to the vessel takeoff (< 2 cm) had a statistically significant effect on endovascular failure. High-grade stenosis of the SMA tended to have a higher probability for frustrated endovascular revascularization. Fortunately, failure of the endovascular approach did not influence patient survival.

Because of the predominantly arteriosclerotic etiology of CMI, patient profiles had typical arteriosclerotic risk factors and were marked by multiple comorbidities. Overall, patients in the ET group had higher perioperative morbidity and rates of PAOD, hyperlipidemia, and nicotine abuse. As described by other authors, they were more frequently pretreated by vascular surgery [7, 11, 15, 17]. Differences in comorbidities can be explained by selection bias in choosing the therapeutic method among the groups in these retrospective studies. Treatment of asymptomatic patients (16.3%) was performed only in selected cases with there was a significant three-vessel disease or poorly collateralized two-vessel occlusions in preparation for an aortic aneurysm repair after careful risk–benefit consideration. Thomas et al. reported that up to 86% of patients with the significant three-vessel disease develop abdominal symptoms or mesenteric ischemia, which is why a prophylactic revascularization should be considered [3]. Because to the limited evidence, the current European guidelines by Terlouw et al. also recommend a “tailor-made approach” in asymptomatic patients with high-grade stenosis of all three mesenteric vessels or in preparation for major abdominal surgery and significant high-grade stenosis of more than two mesenteric vessels after careful consideration of the patient’s overall situation [2].

Indeed, and as has already shown in larger cohort studies, seven patients in our cohort (14%) had symptomatic visceral single-vessel disease [18–20]. As delineated in a review from van Noord et al., a median arcuate ligament syndrome (Dunbar syndrome) in young patients and atherosclerotic disease with restricted collateralization in elderly patients were responsible for clinical symptoms of CMI [19]. In our study, despite the high prevalence of two-vessel disease (67%), a single-vessel revascularization (53%) was performed more frequently. Generally, revascularization of all affected vessels was attempted, especially in OT. The revascularization decision was based on collateralization and technical capabilities. On follow-up examination, two-vessel revascularizations showed no better symptom-free survival than one-vessel revascularization (*P* = 0.75). In a retrospective study (monocentric, 86 open surgery patients), Lejay et al. demonstrated an increased long-term survival for completely revascularized patients (several treated vessels), but also at non-significant level (88% vs. 76%; *P* = 0.54) [20]. Due to the lack of data, it is actually not possible to make a solid recommendation based on the literature concerning the number of vessels to be treated, but a revascularization of all affected vessels might be attempted [2].

Our findings on overall morbidity (22.2%; Table 3) are consistent with those of other published studies from 14 to 40% [7, 20]. Nevertheless, OT is still associated with higher morbidity, which could be expected owing to the extensive nature of the open procedure [7, 9, 21, 22]. This also goes along with the prolonged hospital and intensive care unit stays for the OT group, also associated with higher mortality [1]. The in-hospital mortality in our study was 2.8%, consistent with the 2.6 to 21% that has been reported [17, 23]. As reported in the ESVS guidelines, the difference in in-hospital mortality between the treatment modalities in our study was not significant as well (OT 6.7% vs. ET 0%; *P* = 0.42) [24]. Also, the recent and thus far most comprehensive meta-analysis from Alahdab et al. identified mortality rates for OT that were not significantly higher than those of ET (8% vs. 2%; risk ratio 1.57; 95% confidence interval, 0.84–2.93). These results suggest that the low mortality rates in the highly multi-morbid patient population may reveal a possible publication bias for open procedures [9]. The higher mortality of patients who still needed open surgical revascularization after the endovascular approach, described by Zacharias et al., was inconsistent within the present study [1].

Preoperative laboratory values showed decreased serum protein concentrations (56%) and increased lactate concentration (47.8%), which can be explained by chronic malnutrition [25]. Manifest malnutrition was seen in 50% of the patients, as evidenced by an NRS score of more than 3 [13]. Atkins et al. reported that these deflections were associated with poor survival [15]. Therefore, a patients’ clinical presentation is a particularly relevant prognostic factor for a later outcome. Future efforts should be focused on the early detection of this disease to improve patient outcomes. Preoperatively, attempts to improve nutritional status were not initiated because tonometric studies have shown that oral intake or tube feeding can exacerbate chronic ischemia [26]. Evidence for improving nutritional status via total parenteral nutrition is also flawed. Gatt et al. showed that parenteral nutrition decreases mesenteric blood flow and thus may worsen ischemia [27]. Therefore, revascularization should not be delayed in favor of improving nutritional status [2].

In contrast to the results of other studies, the patency rates between the groups in our study did not differ significantly because of the limited number of patients [1, 21, 28]. Overall, patency rates in the OT group seemed to be higher (100% OT vs. 76.5% ET; *P* = 0.06). However, only patients in the ET group needed revisions during the follow-up period due to recurring symptomatology. In practice, we see no difference in the clinical outcome between the treatment modalities. As reported by Alahdab et al., the 3-year survival did not differ between groups [9]. Also, Tallarita et al. used a propensity score-matched comparison to demonstrate no influence of the type of revascularization (ET vs. OT) on the 5-year survival [23]. Against this background, Lejay et al. recommended a treatment selection based on factors like durability and anatomic characteristics [20]. In fact, the level of evidence is still low, and a comparison of the results is difficult due to the lack of reporting standards. Furthermore, some relevant clinical questions such as the number of vessels to revascularize or the optimal stent type (covered vs. uncovered) are not conclusively clarified and require further randomized studies as well [2, 9].

This study has some limitations. It is a retrospective single-center study, generating bias linked to a retrospective data collection. Furthermore, during the long study period, there has been a progress in medical therapy and endovascular technology that may have affected patients’ outcomes. Finally, there is a preselection bias for OT in this non-randomized study.

## Conclusions

Despite the increasing use of endovascular procedures with better perioperative outcomes, surgical therapy still plays an important role because of its higher technical success and presumably longer durability.

## Data Availability

Not applicable.

## Abbreviations

BMI: Body mass index
CHD: Coronary heart disease
CMI: Chronic mesenteric ischemia
COPD: Chronic obstructive pulmonary disease
CT: Celiac trunk
CTA: Computed tomography angiography
ET: Endovascular treatment
IMA: Inferior mesenteric artery
MRA: Magnetic resonance angiography
OT: Open treatment
PAOD: Peripheral arterial occlusive disease
SD: Standard deviation
SMA: Superior mesenteric artery
